# Eczema and Its Management

**Published:** 1884-06

**Authors:** 


**Eczema and its Management** A practical treatise based on the study of three thousand cases of the disease. By T. DUNCAN BULKLEY, A. M., M. D., Physician to the New York Skin and Cancer Hospital, etc. Second edition. New York: G. P. Putnam's Sons, 27 W. 23d Street London: J. & A. Churchill.

We are heartily glad to receive a new edition of this classical work. Eczema forms at least one-third of all the cases of skin diseases, and it requires no little knowledge and discretion to successfully treat it. Very many of these cases are prolonged and the severity of the disease increased by unwise medication, but there are few of the chronic diseases which respond more happily to careful, thorough and judicious management. This book of Dr. Bulkley is an invaluable guide, practical in every way, logical in arrangement, and exhaustive, for nothing of value is omitted. The publishers have given the book as handsome a presentation as so good a book deserves.

